# Disparities in Postoperative Pain Management: A Scoping Review of Prescription Practices and Social Determinants of Health

**DOI:** 10.3390/pharmacy13020034

**Published:** 2025-02-24

**Authors:** Aidan Snell, Diana Lobaina, Sebastian Densley, Elijah Moothedan, Julianne Baker, Lama Al Abdul Razzak, Alexandra Garcia, Shane Skibba, Ayden Dunn, Tiffany Follin, Maria Mejia, Panagiota Kitsantas, Lea Sacca

**Affiliations:** Charles E. Schmidt College of Medicine, Florida Atlantic University, Boca Raton, FL 33431, USA; asnell2022@health.fau.edu (A.S.); dlobaina2021@health.fau.edu (D.L.); sdensley2022@health.fau.edu (S.D.); emoothedan2022@health.fau.edu (E.M.); jbaker2022@health.fau.edu (J.B.); lalabdulrazz2018@health.fau.edu (L.A.A.R.); alexandragar2021@health.fau.edu (A.G.); sskibba2023@health.fau.edu (S.S.); adunn2023@health.fau.edu (A.D.); tfollin@health.fau.edu (T.F.); mejiam@health.fau.edu (M.M.); pkitsanta@health.fau.edu (P.K.)

**Keywords:** pain management, opioids, healthcare settings, social determinants of health, prescription rates

## Abstract

**Background**: Opioid analgesic therapy has been traditionally used for pain management; however, the variability in patient characteristics, complexity in evaluating pain, availability of treatment within facilities, and U.S. physicians overprescribing opioids have contributed to the current opioid epidemic. Despite large research efforts investigating the patterns of postsurgical pain management and influencing factors, it remains unclear how these overall trends vary across the varying sizes and available resources of academic hospitals, community hospitals, and outpatient surgery centers. The primary aim of this scoping review was to examine the patterns of contemporary postoperative pain management across healthcare settings, including academic medical centers, community hospitals, and outpatient surgery centers. Specifically, this study investigates how prescription practices for opioids, NSAIDs, and acetaminophen are influenced by patient demographics, including sex, race, gender, insurance status, and other social determinants of health (SDoH), to inform equitable and patient-centered pain management strategies. **Methods**: This study utilized The Preferred Reporting Items for Systematic Reviews and Meta-Analyses extension for Scoping Reviews (PRISMA-ScR) and was used as a reference checklist. The Arksey and O’Malley methodological framework was used to guide the review process. To ensure comprehensive coverage, searches were conducted across three major databases: PubMed, Embase, and Cochrane Library. **Results**: A total of 43 eligible studies were retained for analysis. The highest reported Healthy People 2030 category was Social and community context (n = 39), while the highest reported category of SDoH was age (n = 36). A total of 34 articles listed sex and age as SDoH. Additional SDoH examined were race/ethnicity (n = 17), insurance (n = 7), employment (n = 1), education (n = 4), and income (n = 1). This review suggests that there are significant gaps in the implementation of institution-specific, patient-centered, and equitable pain management strategies, particularly in academic hospitals, which our findings show have the highest rates of opioid and NSAID prescriptions (n = 26) compared to outpatient surgical centers (n = 8). Findings from our review of the literature demonstrated that while academic hospitals often adopt enhanced recovery protocols aimed at reducing opioid dependence, these protocols can fail to address the diverse needs of at-risk populations, such as those with chronic substance use, low socioeconomic status, or racial and ethnic minorities. **Conclusions**: Findings from this review are expected to have implications for informing both organizational-specific and nationwide policy recommendations, potentially leading to more personalized and equitable pain management strategies across different healthcare settings. These include guidelines for clinicians on addressing various aspects of postoperative pain management, including preoperative education, perioperative pain management planning, use of different pharmacological and nonpharmacological modalities, organizational policies, and transition to outpatient care.

## 1. Introduction

Postoperative pain management aims to reduce acute postsurgical pain for immediate relief to facilitate a smooth transition for patients when returning to normal function [1]. Inadequate pain control can lead to sensitization, increasing the risk of chronic pain, patient dissatisfaction, delayed mobilization, cardiac and pulmonary complications, and increased morbidity and mortality [1,2,3,4]. Postoperative pain management requires an individualized plan, including consideration of surgical factors, patient factors, and social determinants of health (SDoH), such as age, sex, race/ethnicity, history of chronic opioid use, income or educational status, and other comorbidities. It is dependent on subjective outcomes, like a patient’s perception of their pain severity, tolerance, and analgesic response [1,2,3,4]. Challenges can arise in specific patients, particularly those belonging to vulnerable groups, including pregnant, geriatric, neonatal, opioid-tolerant, or those with a history of substance use disorder [5]. Opioid analgesics have traditionally played a central role in postoperative pain management. However, variability in patient characteristics, the complexity of pain evaluation, and inconsistent access to treatment have contributed to widespread disparities in care. In the United States (U.S.), the overprescription of opioids has fueled the opioid epidemic, highlighting the need for a more balanced approach [1,2,3,4].

In response to rising opioid-related disorders and deaths, contemporary protocols advocate for a multimodal approach to pain relief. This strategy combines pharmacologic therapies, including opioids, NSAIDs, and acetaminophen, to target multiple pain pathways and minimize reliance on opioids [6,7,8]. Despite these risks, opioids remain a cornerstone of postoperative pain management due to their efficacy and history of use [6]. A review of inpatient records found that 80% of patients undergoing low-risk surgery were prescribed an opioid at discharge, and 80% of those prescriptions involved oxycodone or hydrocodone, which are frequently implicated in drug overdose deaths [9]. Though the opioid epidemic has changed institutional attitudes toward pain management, it is unclear the extent to which these changes have impacted practices for postoperative pain management [6]. For example, while a CDC report showed that overall opioid prescriptions peaked in 2010 and steadily decreased through to the study’s conclusion in 2015 [10], a review focused on inpatient postoperative analgesia showed that the rate of prescription of opioids remained stable over the same time period [11]. Further, there is a paucity of research describing how these practices differ across different institutions providing surgical care in the US. Given the significant risks associated with opioid prescriptions and the economic burden of opioid abuse, it is important to investigate whether initiatives aimed at reducing opioid use are being effectively implemented across various surgical care settings in the US, ensuring efficacious treatment while reducing harm.

Healthcare disparities in postoperative pain management are most evident in racial and ethnic minority groups, particularly among lower socioeconomic groups and in individuals with low levels of health literacy [12,13,14,15,16,17]. A meta-analysis of 12 studies showed that cost, socioeconomic status, insurance, and racial identity were the main influential factors affecting the timing and access to adequate care and recovery needs for orthopedic surgery patients from disadvantaged socioeconomic backgrounds [15]. Moreover, several studies highlighted in their findings that patients with private insurance received more expeditious surgical interventions compared to uninsured patients and those with public insurance [18,19,20]. Further, in the setting of acute pain, the odds of Black and Hispanic patients receiving analgesics in the emergency department were significantly lower than White patients [21,22]. In patients over 65 years old and in need of advanced rehabilitation services, White patients were 38% more likely to receive needed rehabilitation care compared to Black patients [21,22]. It was also reported that patients with income levels in the fiftieth percentile or higher were 52% more likely to receive rehabilitation compared to patients below the twenty-fifth percentile of income [21,22]. Hence, pain management care accessibility, affordability, and quality-related challenges remain, particularly when it comes to patients experiencing social determinants of health-imposed disparities. Strategies for equitable practices of postoperative pain management across healthcare settings are essential to ensure access in vulnerable population groups.

Postoperative pain management practices differ across different healthcare settings, such as academic hospitals, community hospitals, and outpatient surgical centers, due to differences in patient demographics, type and volume of procedures performed, and available resources [23]. In academic hospitals, there is a growing emphasis on the implementation of multimodal analgesia that often utilizes NSAIDs or acetaminophen to reduce dependency on opioids, driven by enhanced recovery protocols that aim for quicker patient mobilization and discharge [8]. While effective, these protocols are not widely standardized and have a large degree of heterogeneity in implementation that impacts drug prescriptions and patient outcomes [24]. Comparatively less research exists on the setting of community hospitals, which may aim for the same standards as large academic centers but may vary widely due to slower adoption and standardization of protocols and variation in resources and available training. Outpatient surgical centers have a unique challenge in pain management due to the necessity of immediate patient discharge. In this setting, the use of NSAIDs and acetaminophen is emphasized to avoid complications that could produce an unnecessary hospital admission [25]. Studies have shown that interventions such as The Standardization of Outpatient Procedure (STOP) protocol have been effective in reducing both the prescription of and utilization of opioids without impacting patient-reported average pain in the first seven days [26]. Ultimately, despite large research efforts investigating the patterns of postsurgical pain management and influencing factors, it remains unclear how these overall trends vary between the varying sizes and available resources of academic hospitals, community hospitals, and outpatient surgery centers. Additionally, very few studies have considered the role of SDoH in influencing the access and quality of postoperative pain management services received and the broader consequences this inflicts on underserved communities.

The primary aim of this scoping review is to examine the patterns of contemporary postoperative pain management across healthcare settings, including academic medical centers, community hospitals, and outpatient surgery centers. Specifically, this study investigates how prescription practices for opioids, NSAIDs, and acetaminophen are influenced by patient demographics, including sex, race, gender, insurance status, and other social determinants of health (SDoH), to inform equitable and patient-centered pain management strategies. By identifying variations in prescription patterns, this review aims to address a significant gap in understanding the intersection between healthcare settings and SDoH in postoperative care. The findings from this review are expected to have implications for informing policy recommendations and clinical practices that promote person-centered and equitable pain management strategies.

## 2. Methods

This study utilized The Preferred Reporting Items for Systematic Reviews and Meta-Analyses extension for Scoping Reviews (PRISMA-ScR), which was used as a reference checklist [27]. To guide the review process, the Arksey and O’Malley methodological framework was used [28]. This framework involves five steps: (1) identifying research questions, (2) conducting the literature review, (3) choosing pertinent studies, (4) organizing the data systematically, and (5) compiling, summarizing, and presenting findings. This process ensures transparency and enhances the reliability and validity of the study.

### 2.1. Step 1: Identifying Research Questions

Two research questions were formulated for this study: (1) How does the rate of opioids, NSAIDs, and acetaminophen prescription for postoperative pain management vary between academic hospitals, community hospitals, and outpatient surgical centers? and (2) How are prescription patterns and postoperative pain management practices influenced by patient characteristics and social determinants of health (SDoH)?

### 2.2. Step 2: Conducting the Literature Review

Keywords and MeSH terms for the search strategy were developed in collaboration with a research librarian (TF), an expert in scoping review protocols. The search terms included *postoperative pain management*, *analgesics*, *opioid*, *acetaminophen*, *opiates*, *anti-inflammatory agents*, *social determinants of health*, *sex*, *gender*, *race*, *ethnicity*, *insurance status*, *educational level*, *income level*, *academic medical centers*, *hospitals*, *outpatient clinics*, *community clinics*, *and university medical centers*. To ensure comprehensive coverage, searches were conducted across three major databases: PubMed, Embase, and Cochrane Library. Databases were searched to identify peer-reviewed literature encompassing primary data sources, secondary data sources, and case reports. The literature review was completed over a period of one month, from January 2024 to February 2024. The article screening was carried out by the senior author (LS) and co-authors (AS, DL, SD, EM, JB, LAR, AG, SS, and AD).

### 2.3. Inclusion Criteria

The articles that were included were systematic reviews, meta-analyses, observational, experimental, descriptive, and case-control studies published in English between 2010 and the present. These studies specifically focused on the prescription rates of opioids, NSAIDs, and acetaminophen for postoperative pain management in a range of different settings in adults 18–75 years old. Studies included were carried out in the United States academic hospitals and/or community hospitals and/or outpatient surgical centers.

### 2.4. Exclusion Criteria

Excluded studies encompass case reports, clinical trials, and narrative reviews. Additionally, articles were excluded if they focused on the prescription of opioids, NSAIDs, and acetaminophen that did not pertain to postoperative pain management. Studies conducted in settings other than academic hospitals, community hospitals, or outpatient surgical centers were also not considered. Additional exclusion criteria were articles focusing on patients aged over 75 or under 18 years, articles published before 2010, studies conducted outside the United States, and non-English language articles.

### 2.5. Step 3: Selecting Studies Relevant to the Research Questions

All student co-authors (AS, DL, SD, EM, JB, LAR, AG, SS, and AD) extracted and summarized data from the relevant articles. The senior author (LS) reviewed all tabulated data to resolve any discrepancies. Initial categorization of data was conducted by co-authors (AS, DL, SD, EM, JB, SS, and AD) and was compared to independent categorization with reviewer pair (LAR and AG) and senior author (LS) for inter-rater reliability in a subsample of 80% (n = 34) of the studies. Inter-rater reliability was conducted in two rounds with discrepancies resolved by consensus discussion. The resulting inter-rater reliability was 90% for all data tabulated.

Summary tables included one evidence table describing study characteristics, types of SDOH, and outcomes. First, types of SDOH were listed and organized based on the five categories described by Healthy People 2030, which are Economic Stability, Education Access and Quality, Health Care Access and Quality, Neighborhood and Built Environment, and Social and Community Context [29]. The Healthy People 2030 is a set of science-based objectives with goals to observe progress and encourage and focus action [29]. Following the World Health Organization’s (WHO) call to address SDOH to preserve health and quality of life, The Healthy People 2030 first introduced SDOH objectives in 2010 [29].

Variance in rates of prescription of Opioids, NSAIDs, and Acetaminophen for postoperative pain management were reported (Table 1). Table 2 highlights the prevalence of prescription of opioids, NSAIDs, and acetaminophen for postoperative pain management across Academic Hospitals, Community Hospitals, and Outpatient surgical centers to focus on differences among these centers.

### 2.6. Steps 4 and 5: Charting the Data and Collation, Summarization, and Reporting of Results

Study characteristics were tabulated for primary author/year, study design, sample size, study population, age range, study purpose, type of SDOH, SDOH category based on HP 2030, type of postoperative pain, type of prescription, and variance in prescriptions rates based on SDOH (Table 1). Identified articles were arranged based on primary author/year, type of surgery, type of prescription, type of postoperative pain, and healthcare setting (Table 2).

## 3. Results

**Figure 1:**
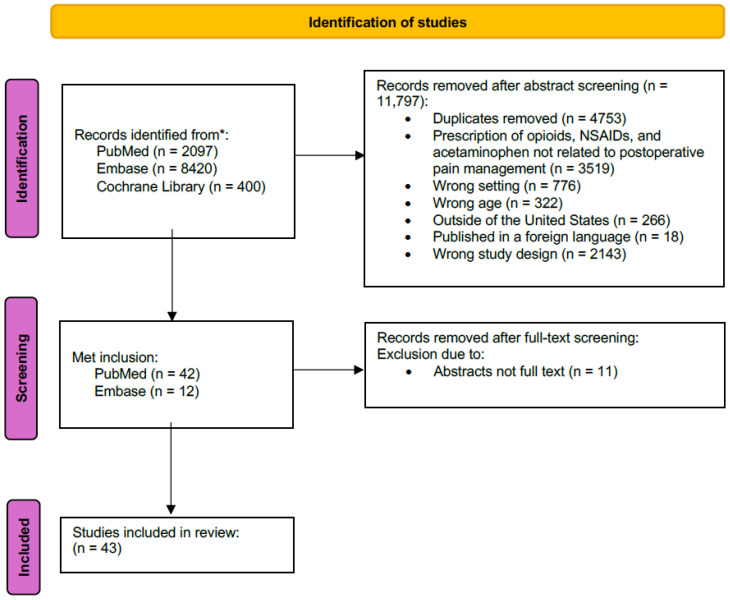
PRISMA flow chart. * No tracing of references or grey literature was included in the identification process.

The initial study extraction generated 11,851 studies from PubMed (n = 2097), Embase (n = 8420), and Cochrane Library (n = 400). Studies were excluded if they had to do with the prescription of opioids, NSAIDs, and acetaminophen not related to postoperative pain management (n = 3519), were the wrong setting (n = 776), wrong age (n = 322), outside of the United States (n = 266), published in a foreign language (n = 18), or wrong study design (n = 2143). Duplicated studies were removed (n = 4753). A total of 54 studies met the inclusion criteria from PubMed (n = 42) and Embase (n = 12). An additional 11 studies were excluded following a full study review due to being abstracts and not full text. A total of 43 eligible studies were retained for analysis (Figure 1). The retained studies were published between 2016 and 2023. Most studies (n = 31/43, 72%) were published in the last 5 years. Study designs included retrospective cohort studies (n = 25), prospective cohort studies (n = 8), cross-sectional studies (n = 5), prospective-retrospective cohort studies (n = 2), retrospective case series (n = 1), systematic review (n = 1), and retrospective chart review (n =1). Sample size ranged from n = 58 to n = 852,111. The healthcare settings included academic hospitals (n = 26), outpatient surgical centers (n = 8), both academic medical centers and outpatient surgical facilities (n = 5), research databases (n = 2), and academic community hospitals (n = 2).

### 3.1. SDOH Explored and Classification Based on the Healthy People 2030 Categories

**Figure 2:**
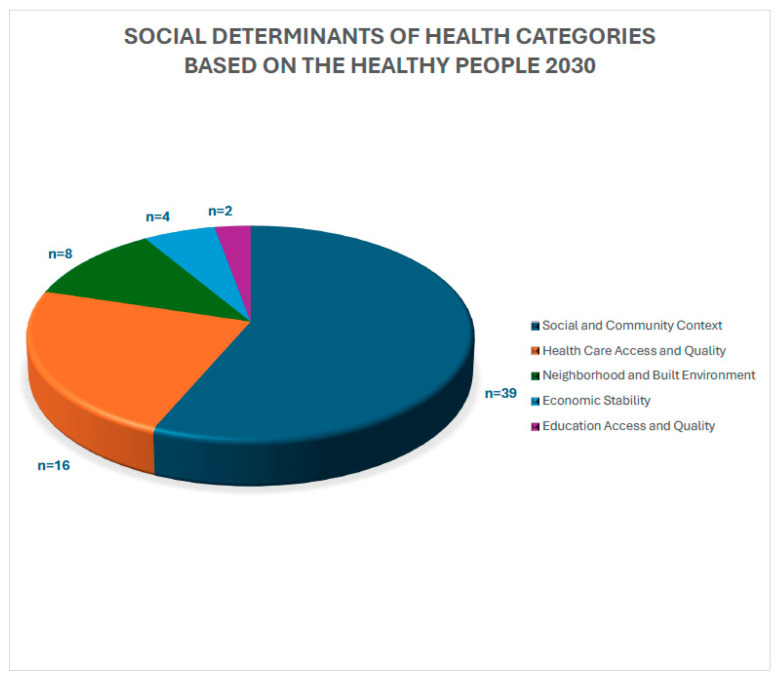
Classification of social determinants of health based on the Healthy People 2030 five domain categories.

**Figure 3:**
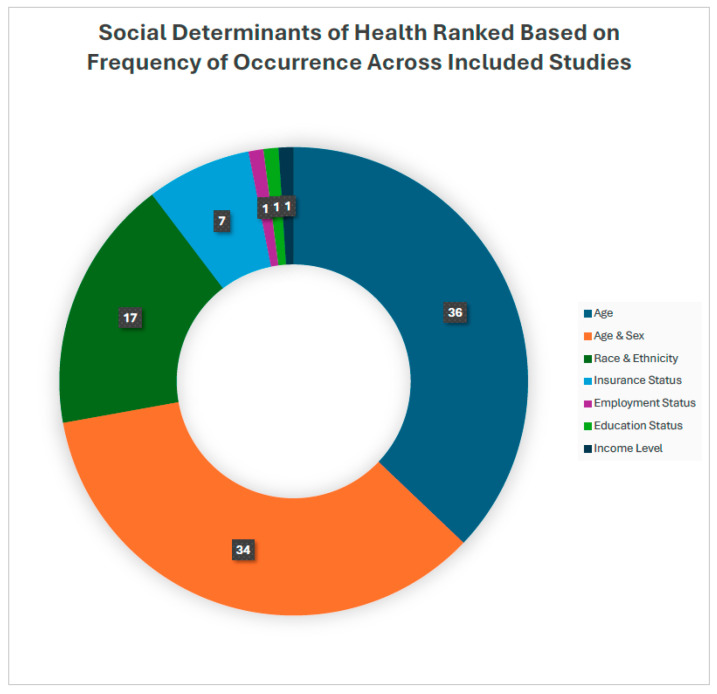
Frequency of reported social determinants of health in postoperative pain management practices.

The Healthy People 2030 classification identifies public health priorities to help individuals, organizations, and communities across the United States improve health and well-being. Healthy People 2030 is the organization’s fifth version and was based on 40 years of knowledge and data. Their social determinants of health are defined by five categories: Economic stability, Education Access and Quality, Health Care Access and Quality, Neighborhood and Built Environment, and Social and Community Context. Economic stability focuses on employment programs, career counseling, and childcare opportunities to help people achieve steady employment. The education access and quality objective focuses on helping children do well in school and providing higher quality education. Healthcare access and quality focuses on improving healthcare insurance coverage rates, accessibility to primary care providers, and preventative care. The neighborhood and built environment objective seeks to help improve health and safety in the community through advocacy work at the local, state, and federal levels of government. Finally, social and community context focuses on social support by helping people find safe spaces in their communities. Based on these categories, the SDOH examined in this study were categorized based on the Health People 2030 categories. Healthy People 2030 categories reported by high to low frequency of occurrence were Social and community context (n= 39), Health care access and quality (n = 16), Neighborhood and Built Environment (n = 8), Economic Stability (n = 4), and Education Access and Quality (n = 2) (Figure 2). The highest reported category of SDoH was age (n = 36). A total of 34 articles listed sex and age as SDoH (Table 1). Additional SDoH examined were race/ethnicity (n = 17), insurance (n = 7), employment (n = 1), education (n = 4), and income (n = 1) (Figure 3).

### 3.2. Variance in Prescription Rates Based on Healthcare Setting and SDoH

**Figure 4:**
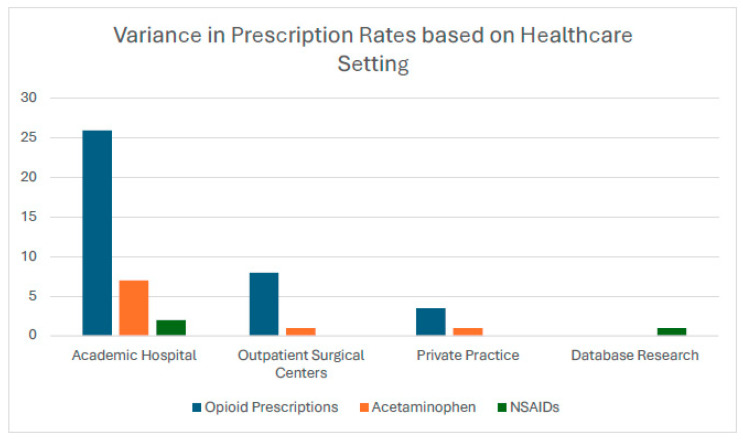
Type of prescriptions based on healthcare setting.

The highest rates of opioid prescriptions were reported in academic hospitals (n = 26) compared to outpatient surgical centers (n = 8). Other types of prescriptions included acetaminophen (n = 9; academic hospital (n = 7), outpatient surgical center (n = 1), and private practice/academic hospital (n = 1)) and NSAIDs (n = 3; academic hospital (n = 2) and database research (n = 1)) (Figure 4). A total of 24 out of 43 studies (Table 2) highlighted findings regarding the variance in opioid prescription rates based on SDoH, considering factors such as age, sex, race, income, insurance type and status, substance use, geographic location, education, and settings of care. Among the studies evaluating age, 11 examined its relationship with opioid use. Four studies reported no significant association between age and opioid prescriptions [41,54,64,70]. Conversely, seven studies indicated that younger patients were associated with higher rates of opioid use [35,37,55,59,62,68,69].

In terms of sex, six studies investigated its impact on opioid prescriptions. Three studies found that males received higher opioid prescriptions compared to females [35,37], while one study indicated that female patients were more likely to be prescribed opioids [62]. However, Locketz et al. [54], Zipple et al. [70], and Qian et al. [64] reported no significant differences based on sex. Regarding race, five studies analyzed its association with opioid use. Three studies found no correlation between race and opioid prescriptions [57,59,70]. However, Letchuman et al. [53] reported that White patients received higher opioid doses than Black and Asian patients of similar demographics, while Bronstone et al. [36] highlighted a trend of high opioid prescriptions among Black patients. Income and insurance type were also evaluated in six studies (Table 2).

Waljee et al. [68] and Cooperman et al. [37] observed a negative correlation between income and opioid prescriptions, indicating that patients in higher-income areas were less likely to receive opioid refills. Conversely, O’Sullivan et al. [59] found that patients with Medicaid or Medicare were less likely to receive opioid prescriptions compared to those with private insurance. Additionally, Kim et al. [50] noted that self-pay patients and those on Medicaid tended to consume higher amounts of opioids. Lutsky et al. [56] reported that patients with commercial insurance utilized more opioids intraoperatively but filled fewer prescriptions postoperatively. In contrast, Bhashaym et al. [35] found no significant differences in opioid prescriptions based on insurance type. Substance use history was another important factor, with studies indicating that patients with a preoperative history of substance use or substance use disorders required higher opioid dosages [35,55,62]. As-Sanie et al. [31] further demonstrated that patients with chronic pain on medication needed greater opioid doses postoperatively. Keller et al. [48] examined the effects of geographic location and education, finding that patients in the Western U.S. exhibited higher rates of new persistent opioid use, whereas those with a Bachelor’s degree or higher had lower rates of opioid use. Lastly, in regard to care settings, Nouraee et al. [58] reported that fewer pills were prescribed and consumed in outpatient settings compared to inpatient ones (Table 2).

### 3.3. Types of Surgery of Postoperative Pain

The reviewed studies described a wide range of surgeries associated with acute and chronic postoperative pain. Types of surgeries included orthopedic, gynecologic, abdominal, cardiovascular, and otolaryngologic procedures. Acute postoperative pain was reported in the majority of studies (n = 41) following surgery completion, including laparoscopic cholecystectomy (n = 3) [30,41,66], total hip and knee arthroplasty (n = 2 for each) [51,58,59,62], bariatric surgeries such as sleeve gastrectomy and Roux-en-Y gastric bypass (n = 1) [39], otolaryngology procedures including middle ear and sinus surgeries (n = 2) [37,54], orthopedic procedures like ACL reconstruction and rotator cuff surgery (n = 3) [36,40,59], and cesarean sections (n = 2) [34,57]. Chronic postoperative pain was reported less (n = 3) but was observed in specific procedures, including spinal fusion (n = 2) [60,67], dialysis access surgery (n = 2) [45,65], and cytoreductive surgery (n = 1) [61] (Table 2).

## 4. Discussion

This scoping review explored postoperative pain management across various healthcare settings and how prescription practices for pain medications vary based on patient characteristics such as sex, race, gender, and insurance status. By examining variations in prescription rates and patterns, the study sought to address gaps in understanding how different healthcare settings and SDoH intersect in postoperative care, ultimately guiding policy recommendations and clinical practices for more personalized and equitable pain management strategies.

The highest reported SDoH in this paper was age, followed by sex. SDOH constitutes 80–90% of the modifiable factors that influence public health, which is not limited to surgical access and postoperative recovery [71]. All patients, regardless of race, ethnicity, sex, age, or socioeconomic status (SES), should receive adequate pain management after surgery to ensure health equity [71]. However, recent studies have noted healthcare disparities in postoperative pain management [72,73]. One theory for the observed differences in treatment among patients is the presence of healthcare providers’ unconscious biases toward patients [72,74]. In the context of older adults, providers might have concerns about adverse effects when prescribing opioids in elderly populations, ultimately leading providers to overlook patients’ pain management needs [74,75]. Rambachan et al. [76] found that older, hospitalized general medicine patients from minoritized groups with geriatric conditions are at risk for inequitable pain assessment, resulting in pain being under-assessed and inadequately treated. Similarly, this trend was seen in females and individuals with low socioeconomic status (SES), such as those with low household income, poor insurance status, or low level of education [72]. Furthermore, being a female was significantly associated with lower amounts of opioid administration after cardiac surgery [77]. Nevertheless, optimal postoperative pain relief has been a challenge for racial minorities and those with lower SES; for instance, although African American and Hispanic individuals reported a significantly higher pain intensity score, they were prescribed fewer opioids than their non-Hispanic White counterparts [72,73]. Language barriers between individuals and providers can perpetuate the low quality of care by either stemming from the provider’s cultural preconception regarding the patient’s pain tolerance or anticipated additional time required for pain assessment [74]. This disparity has shown that patients with limited English proficiency have a reduced propensity to request medication refills after discharge, contributing to diminished access to postoperative care [78]. Standardization of care is needed to reduce disparities in postoperative pain management [72].

Limited healthcare access, lack of insurance coverage, and prior or current substance use history have been significant barriers to opioid prescriptions and effective pain management, and the consequences of such disparities can lead to worsening the burden of the opioid epidemic on the healthcare system [79,80]. These findings further reiterate the importance of the highest ranking SDoH domain, the social and community context, highlighted in the Healthy People 2030 objectives as a crucial factor to consider in building an equitable context for the delivery of postoperative pain management practices across eclectic healthcare settings. Studies have revealed that Hispanic individuals are more likely to have self-pay visits for pain management compared to other populations, which might be a reflection of Hispanics being one of the largest uninsured groups in the US [79,81,82]. Having a lack of insurance coverage may lead providers to be hesitant with opioid management due to there being a need for frequent visits and monitoring [79]. Furthermore, long-acting opioids often face stricter regulations, and insurance coverage for these medications is less likely [83]. It has been noted that pharmacies in areas of non-White neighborhoods tend to have fewer opioid prescriptions available, which, when compounded with limited insurance coverage or lack of insurance, leads to inequities in pain management [79,83,84]. Other disparities associated with opioid prescription include prior substance use history, in which patients have described many negative feelings of stigmatization and discrimination, with clinicians labeling them as “drug addicts”, dismissing their pain, or clinicians being hesitant or completely refusing care for the patient’s pain [80]. Chronic pain is a major factor for increased opioid consumption, which emphasizes the need for adequate pain control after an operation and should not be withheld from patients with an active or prior substance use history because of the fear of worsening addiction or triggering a relapse [8,85]. For patients with opioid use, a higher dose is likely needed after surgery, and it has been noted that poorly treated pain can trigger relapses [8,85]. Moreover, interventions should focus on national medical policies, insurance, and healthcare systems, and providers are required to minimize discriminatory practices, with considerations of consultative expertise with pain specialists when it comes to postoperative pain management in patients with a substance use history [8,80,85].

Our review suggests that there are significant gaps in the implementation of institution-specific, patient-centered, and equitable pain management strategies, particularly in academic hospitals, which our findings show have the highest rates of opioid and NSAID prescriptions (n = 26) compared to outpatient surgical centers (n = 8). Findings from our review of the literature demonstrated that while academic hospitals often adopt enhanced recovery protocols aimed at reducing opioid dependence, these protocols can fail to address the diverse needs of at-risk populations, such as those with chronic substance use, low socioeconomic status, or racial and ethnic minorities. For example, insurance status and economic stability are important factors; patients with Medicaid or Medicare were found less likely to receive prescriptions compared to those with private insurance, which could leave vulnerable groups under-treated for pain [30]. Further, hospital-level factors influencing these disparities include differences in hospital case mix, trainee prescribing behaviors, and lack of standardized opioid prescribing guidelines, particularly in teaching environments [60]. These findings underscore the importance of implementing evidence-based, procedure-specific prescribing protocols to promote safer practices and reduce the risks associated with postoperative opioid use.

Although the focus of this study was hospital factors, in particular teaching hospital status, our results show that patient-level factors such as SDoH and procedure type contribute significantly to variation in postoperative opioid prescribing. The effects of some SDoH are not always unidirectional; when considering race, Letchuman et al. [64] found that White patients received more opioid prescriptions than Black and Asian patients of similar demographics, while Bronstone et al. [62] identified disproportionately high opioid prescription rates among Black patients. These findings highlight that postoperative pain management is influenced by SDoH but also that these effects are not consistent but instead are highly patient and setting-dependent. Additionally, inpatient surgical procedures in academic hospitals had the highest reported rates of opioid prescriptions for postoperative pain management. Disparities in pain management not only perpetuate inequities but also strain the healthcare system by contributing to suboptimal pain control, increased readmissions, and a heightened risk of chronic pain syndromes, underscoring the urgent need for tailored, equitable strategies to improve outcomes across all healthcare settings [86,87].

Moreover, a significant gap remains in the dissemination and implementation of clinical practice guidelines to promote evidence-based, effective, and safer postoperative pain management in children and adults. In 2016, the American Pain Society (APS), with input from the American Society of Anesthesiologists, commissioned an interdisciplinary expert panel to develop such a type of guideline to provide recommendations that addressed various aspects of postoperative pain management, including preoperative education, perioperative pain management planning, use of different pharmacological and nonpharmacological modalities, organizational policies, and transition to outpatient care [8]. Initially drafted based on a systematic review, the recommendations highlight that optimal pain management should be initiated in the preoperative period to be able to culturally tailor the plan of care to reflect not only the patient’s specific needs [8]. However, out of the thirty-two recommendations, only four were supported by high-quality evidence, while eleven were formulated based on low-quality evidence. This is concerning since the majority of recommendations are in the areas of patient education and perioperative planning, patient assessment, organizational structures and policies, and transitioning to outpatient care. These gaps align with the findings from a comprehensive review examining pharmacologic and non-pharmacologic pain management strategies, evaluating their effectiveness, and identifying inconsistencies and gaps in current practices in emergency departments (ED) [88]. Key challenges in the ED environment consisted of time constraints, variability in clinical protocols, and challenges in addressing diverse patient populations, such as tailoring standardized plans to fit the needs of pediatric, geriatric, and chronic pain patients. Recommendations from highlighted studies include the importance of having standardized pain assessment tools and protocols to improve consistency in pain management, integration of innovative technological advances and multimodal approaches to enhance pain management practices, and improved training opportunities for ED staff [88]. Furthermore, enhanced patient education, active involvement in care, individualized non-pharmacological pain relief methods, and PCA use improve perceived pain relief and satisfaction with pain management, particularly when it comes to community healthcare settings in resource-constrained areas [89]. Therefore, the development of more effective and uniform pain management practices in healthcare ultimately leads to better patient outcomes and experiences; however, the continuous striving for ongoing research and adaptation of best practices to meet the evolving needs of patients in the community and academic healthcare settings is essential.

### Limitations

Limitations of this study include the exclusion of case reports, case studies, and literature reviews from ‘grey literature’, which potentially limits the breadth of data collection. Our study was also limited in scope by not including tracing of reference lists from included studies, although a comprehensive search of relevant psychosocial databases was conducted involving an initial, secondary, and tertiary screening led by the senior author and co-authors. Further, while search terms used were intentionally broad to account for the diversity of surgical subspecialties in postoperative pain management, the unexpected omission of specific terms may have occurred. This risk was mitigated by collaboration with an expert librarian who developed a rigorous protocol and search strategy for this study. Moreover, our review was limited in scope to studies published in English solely and conducted in the United States. Future studies should broaden the search strategy to include global settings for a worldwide comparison of opioid prescription rates. Finally, the extraction of SDOH proved challenging, as most studies referred to these variables as sociodemographic factors rather than explicitly as SDOH. Future research should advocate for standardizing the nomenclature used for these determinants in the context of opioid prescriptions for postoperative pain management to enhance clarity and comparability. This, in turn, will allow further examination of the development of culturally sensitive and patient-centered approaches to pain management that account for socioeconomic and cultural differences.

## 5. Conclusions

In an era with a heightened focus on pain management, this review sheds light on the impact of SDoH on the continued variability of postoperative pain management across healthcare settings. Our findings emphasize the critical need for institution-specific, personalized, and equitable strategies that account for SDoH impacting patient needs. This, in turn, ensures enhanced access to individual-specific tailored pain management plans that take into account not only the patient’s medical needs but also their socioeconomic status, which affects access, quality, and affordability of pain management options received. Future research should focus on tailoring pain management protocols to specific populations and care settings, assessing their impact on both patient outcomes and system-wide efficiency. Refining these protocols will help establish a foundation for more effective, equitable, and optimal approaches to postoperative pain management. Researchers should also investigate the role of emerging digital health tools, such as telemedicine and mobile apps, in reducing disparities in postoperative pain management due to their effective and feasible reach and impact, along with their continuously evolving role in the delivery of healthcare services.

## Figures and Tables

**Table 1:** Study characteristics of included studies.

#	Primary Author/Year	Study Design	Sample Size	Study Population	Age Range	Study Purpose	Type of SDOH	Category Based on HP 2030
1	Allan et al., 2020 [30]	Prospective-Retrospective Cohort Study (Quality Improvement Project)	n = 133	Patients who underwent one of the following elective outpatient procedures: laparoscopic cholecystectomy, laparoscopic inguinal hernia repair, laparoscopic umbilical hernia repair, open umbilical hernia repair, and laparoscopic inguinal hernia repair in the University of Iowa hospital system.	>18 years old	To examine postoperative pain prescriptions for outpatient procedures in a university hospital system	Age, Sex	Social and Community Context
2	As-Sanie et al., 2017 [31]	Prospective Cohort Study (Quality Improvement Project)	n = 102	English-speaking patients undergoing hysterectomy for benign, non-obstetric indications at a university hospital between August and December of 2015	>18 years old	To quantify physician prescribing patterns and patient opioid use in the two weeks after hysterectomy at an academic institution, and determine whether patient factors predict postsurgical opioid use and pain recovery	Age, Race, Access to Health Services	Social and Community Context
3	Asanad et al., 2020 [32]	Cross-Sectional Study	n = 136	Urologists performing vasectomies in men identified as having undergone clinic vasectomy between March 2018 and May 2019 without surgical or postsurgical complications	Median: 60.5 years old	To determine urologist opioid prescribing patterns and patients’ pain control medication regimens (opioid and anti-inflammatory) after vasectomy	Sex, Age	Social and Community Context
4	Asmaro et al., 2021 [33]	Retrospective Cohort Study	n = 203	Patients who were discharged home following a craniotomy for brain tumor resection between June 2017 and December 2018 at Henry Ford Hospital and Henry Ford West Bloomfield Hospital	>18 years old	To evaluate the effectiveness of a system-wide campaign to reduce opioid prescribing excess while maintaining adequate analgesia	Age, Sex, Race	Social and Community Context
5	Bateman et al., 2017 [34]	Cross-Sectional Study	n = 720	Women who received a cesarian section at one of six academic medical centers in the United States from September 2014 to March 2016	>18 years old	To define the number of opioid analgesics prescribed and consumed after discharge after cesarian delivery	Age, Sex, Race, Insurance Type	Healthcare Access and Quality
6	Bhashaym et al., 2019 [35]	Prospective Cohort Study	n = 303	Patients of an orthopedic surgery practice undergoing one of the following procedures: Arthroscopy/tendoscopy, Neuroma, Hardware removal, Sesamoidectomy, Mass or ganglion resection Lateral ligament reconstruction, Peroneal tendon repair, Bunion surgery, First MTP fusion/cheilectomy, Forefoot procedures, Fusions (except first MTP), Total ankle replacement, Flatfoot correction, Below-knee amputation Open reduction internal fixation	>18 years old	To prospectively assess opioid consumption patterns following implementation of prescription guidelines in patients undergoing outpatient foot and ankle surgery	Age, Sex, Insurance Type	Healthcare Access and Quality
7	Bronstone et al., 2022 [36]	Retrospective Cohort Study (electronic medical record (EMR) review)	n = 655	Adults who underwent one of the five most common outpatient lower extremity orthopedic surgeries performed during 2013 to 2018 at an urban tertiary care academic medical center	>18 years old	To examine trends in opioid prescribing for common outpatient lower extremity orthopedic surgeries in a population vulnerable to prolonged opioid use before and after the enactment of a 2017 Louisiana state law limiting opioid prescribing for acute pain	Sex, Race/Ethnicity, Insurance status, Access to Healthcare services, Policies	Healthcare Access and Quality, Neighborhood and Built Environment, Social and Community Context
8	Cooperman et al., 2017 [37]	Retrospective Cohort Study	n = 17,431	Adult opioid-naïve outpatients who have undergone middle ear or mastoid surgery from 2005 to 2017 identified by ICD-9-CM, ICD-10-CM, and CPT-4 codes	>18 years old	To describe opioid stewardship in ambulatory otologic surgery from 2005 to 2017	Sex, Race, Age, Income level, Education, Region	Economic Stability, Education Access and Quality, Healthcare Access and Quality, Social and Community Context, Neighborhood and Built Environment
9	Cron et al., 2019 [38]	Retrospective Cohort Study	n = 17,075	Adult patients with age 18 to 64 who underwent the following non-emergent surgical procedures between January 2012 and June 2016	18–64 years old	To compare opioid prescribing across hospitals within a large statewide quality collaborative to compare the number of opioids prescribed, as well as high-risk patterns of opioid prescribing, at teaching hospitals compared to non-teaching hospitals. To identify other hospital factors associated with number of opioids prescribed postoperatively	Sex, Age, Access to Health Services	Healthcare Access and Quality, Social and Community Context
10	Ford et al., 2022 [39]	Retrospective Cohort Study/Cross-Sectional Survey Study	n = 192	Patients who underwent elective bariatric surgery at a single academic institution from April2018 to March 2019	>18 years old	To define the current prescribing guidelines at Medical College of Wisconsin for opioid medications following elective bariatric surgery and determine the actual opioid utilization after surgery	Age, Sex, Race/Ethnicity	Social and Community Context
11	Fujii et al., 2018 [40]	Retrospective medical record; prospective telephone questionnaire and medical record data	n = 10,112; n = 539	**Retrospective data:** Patients 18 years and older who were discharged to home after one of 19 procedures in general surgery, orthopedic surgery, urology, and gynecology, during the study period; **Prospective data:** patients who underwent one of 13 common procedures in general surgery, orthopedic surgery, or urology during the recruitment period.	>18 years old	To identify opioid prescribing and use patterns after surgery to inform evidence-based practices	Age, Sex, Insurance type	Healthcare Access and Quality, Social and Community Context
12	Hill et al., 2017 [41]	Retrospective Cohort Study/Cross-Sectional Survey Study	n = 581	Only opioid naïve patients, defined as having no history of opioid use within the 30 days before their procedure, who underwent either 5 most common outpatient general surgery procedures performed at our academic medical center in 2015	>18 years old	To examine opioid prescribing patterns after general surgery procedures and to estimate an ideal number of pills to prescribe	Age, Access to Health Services	Healthcare Access and Quality, Social and Community Context
13	Hite et al., 2023 [42]	Observational, Cross-Sectional Survey Study	n = 104	Patients undergoing outpatient anorectal surgery from January to May 2018 by the colorectal service at the Vanderbilt University Medical Center’s tertiary care medical center	>18 years old	To evaluate patient opioid use and create prescribing recommendationsfor anorectal surgery	Age, Sex, Race, Employment	Economic Stability, Social and Community Context
14	Holst et al., 2020 [43]	Prospective Cohort Study/Cross-Sectional Survey Study	n = 153	Patients undergoing lung resection with MIS or thoracotomy from 3 academic centers within our hospital system from 13 March 2017 to 19 January 2018.	Median: 64 years old (MIS) and 66 years old (thoracotomy)	To investigate post-discharge opioid use in patients after hospitalization for surgical lung resection with a focus on the relative amount of opioid prescribed and the disposition of unused opioid	Age, Sex, Race, Access to Health Services	Healthcare Access and Quality, Social and Community Context
15	Jandali et al., 2020 [44]	Retrospective Cohort Study	n = 185	Patients getting major oral cavity resection, oropharynx resection, laryngectomy, or pharyngectomy between July 2017 and January 2019	18–75 years old	To evaluate narcotic usage and length of stay, in addition to several other outcomes, following the implementation of an ERAS protocol	Age, Sex	Social and Community Context
16	Janek et al., 2022 [45]	Retrospective Cohort Study	n = 58	Patients undergoing dialysis access surgery (AVF, AVG, PD) from August 2018 to January 2019 at UW-affiliated hospitals	18–75 years old	To characterize opioid pain medication use following dialysis access surgery to promote a conservative approach to postoperative opioid prescription	Age, Sex	Social and Community Context
17	Jawad et al., 2022 [46]	Cross-Sectional Study	n = 3152	Patients undergoing any of the 41 most common otolaryngology procedures with the department of otorhinolaryngology and communication sciences at Ochsner medical center between January 2013 and December 2017	>18 years old	To investigate opioid prescribing patterns for otolaryngology procedures at a tertiary hospital with the aim of characterizing postoperative pain and opioid use	Age, Sex	Social and Community Context
18	Katz et al., 2021 [47]	Retrospective Cohort Study	n = 561	All adult patients undergoing common outpatient otolaryngology surgical procedures in the 3 months before enactment of HB21 and 3 months after enactment of HB21	>18 years old	To determine changes in the prescriptions of postoperative opioids in response to Florida state legislation restricting the number of days for which these medications could be prescribed to 3 days in most circumstances or 7 days at provider discretion	Age, Sex	Social and Community Context
19	Keller et al., 2021 [48]	Retrospective Cohort Study	n = 23,426	Opioid naïve patients who underwent outpatient hemorrhoid, fissure, or fistula procedure from January 2010 to June 2017 using optums deidentified clinformatics database	>18 years old	To determine the prescribing trends, new persistent opioid use rates, and factors associated with NPOU after ambulatory anorectal procedures	Age, Sex, Race, Education, Geographic Region	Social and Community Context, Neighborhood and Built Environment
20	Kelley et al., 2020 [49]	Descriptive Cross-Sectional Study	n = 104	Urology residents	>18 years old	To evaluate and characterize postoperative opioid prescribing habits and trends among urology residents in the United States	Sex, Race/Ethnicity, Geographic Region	Education Access and Quality, Social and Community Context, Neighborhood and Built Environment
21	Kim et al., 2016 [50]	Prospective Cohort Study	n = 1416	Patients undergoing upper extremity surgery between April 2014 and October 2014 at a single private academic group	18–93 years old	To prospectively evaluate opioid consumption following outpatient upper-extremity surgical procedures to determine opioid utilization patterns and to develop prescribing guidelines	Age, Sex, Insurance Type	Healthcare Access and Quality, Social and Community Context
22	Kirkness et al., 2013 [51]	Retrospective Cohort Study	n = 115	Patients who were 40+ years old at the time of their total hip arthroplasty	>40 years old	To evaluate pharmacotherapy strategies for managing postoperative pain in patients undergoing total hip arthroplasty in a real-world clinical setting	Sex, Age, Geographic region	Social and Community Context, Neighborhood and Built Environment
23	Kulik et al., 2015 [52]	Retrospective Cohort Study	n = 277,576	Patients who, during the course of hospital stay, underwent a coronary artery bypass graft operation between 2004 and 2010	>50 years old	To examine the utilization patterns of NSAIDs before and after the FDA advisory for coronary artery bypass graft procedures and evaluate the predictors of postoperative NSAID administration early after coronary artery bypass graft procedures	Sex, Age, Policy	Social and Community Context, Neighborhood and Built Environment
24	Letchuman et al., 2022 [53]	Retrospective Cohort Study	n = 1944	Opioid-naive patients 18 years and older who underwent a spine surgical procedure, had a postoperative length of stay of at least 24 h, and who were discharged to home, a skilled nursing facility, or a rehabilitation facility	52–72 years old	To characterize the perioperative opioid requirements across racial groups after spine surgery	Race/Ethnicity	Social and Community Context
25	Livingston-Rosanoff et al., 2020 [21]	Retrospective Cohort Study, Prospective Cross-Sectional Survey	n = 627	Adult patients who underwent an outpatient anorectal procedure performed by one of six staff colorectal surgeons at the University of Wisconsin between January 2018 and September 2019	>18 years old	To characterize opioid prescribing and use among patients undergoing outpatient anorectal procedures and to assess adequacy of postoperative pain management	Sex	Social and Community Context
26	Locketz et al., 2019 [54]	Prospective Cohort Study	n = 219	Patients undergoing nasal or sinus surgery (including septorhinoplasty, septoplasty, functional endoscopic sinus surgery, inferior turbinate reduction, and/or dacryocystorhinostomy)	Mean age 48.2 years old	To understand opioid prescribing patterns and postoperative consumption following sinonasal surgery by surveying postoperative patients regarding their postoperative pain and use of prescribed opioid pain medication	Age, Sex, History of Smoking	Social and Community Context, Access to Healthcare Services
27	Long et al., 2018 [55]	Retrospective Case Series	n = 237	Patients who had thyroid and parathyroid surgery performed between January 2014 and August 2016 by a single surgeon	Mean age: 52.9 years old	To characterize the prescribing patterns of opioids by a single surgeon and the nature of pain and opioid consumption following thyroid and parathyroid sur- gery	Age, Sex	Social and Community Context
28	Lutsky et al., 2020 [56]	Retrospective Cohort Study	n = 269	Patients undergoing elbow, wrist, and hand surgery by two hand surgeons at one academic outpatient surgical center	16–88 years old	To assess postoperative opioid, sedative, and benzodiazepine usage in a Medicare population after hand surgery	Age, Sex	Social and Community Context
29	Matteson et al., 2023 [22]	Systematic Review	n = 35 studies	Individuals who underwent gynecologic surgery of any type for benign indications (including hysterectomy of any route, laparoscopy without hysterectomy, pelvic organ prolapse repair, incontinence surgery, hysteroscopy, dilation and curettage, and cervical conization)	>18 years old	To summarize the amount of outpatient opioid medication used after gynecologic surgery and assess the incidence of new persistent opioid use and misuse after gynecologic surgery	Age, Sex, Race, Employment, Insurance type, Access to Healthcare Services	Healthcare Access and Quality, Social and Community Context
30	McKinnish et al., 2021 [57]	Retrospective Cohort Study	n = 268	Women undergoing vaginal and cesarean delivery from July–October 2018 with an active opioid order as an inpatient	22–33 (women of childbearing age)	To investigate the effect of race on inpatient postpartum opioid consumption and the number of opioids prescribed at discharge after vaginal cesarean delivery	Race/Ethnicity	Social and Community Context
31	Nouraee et al. 2022 [58]	Retrospective Chart Review	n = 925	Patients who underwent elective primary total knee arthroplasty and total hip arthroplasty in one of five ambulatory surgical centers or within one of five inpatient area hospitals from August 2018–2019	Mean age: 56.8 years old	To compare patients who underwent total knee arthroplasty or total hip arthroplasty in an outpatient and inpatient setting and determine postoperative opioid prescription and consumption, pain, and satisfaction with pain control	Age, Sex, Access to Health Services	Healthcare Access and Quality, Social and Community Context
32	O’Sullivan et al., 2023 [59]	Retrospective Cohort Study	n = 12,366	Patients who underwent one of the eight most common orthopedic procedures (rotator cuff repair, shoulder arthroscopy, knee arthroplasty, meniscus repair, knee articular cartilage repair, carpal tunnel repair, total hip arthroplasty, or total knee arthroplasty) between January 2017 and March 2021	>18 years old	To determine whether Black, Hispanic/Latino, or Asian/Pacific Islander patients are less likely than non-Hispanic White patients to receive an opioid prescription after an orthopedic procedure and, if they do receive a prescription, do they receive a lower analgesic dose	Race/Ethnicity, Sex, Age, and Insurance Status	Social and Community Context, Economic Stability, and Healthcare Access and Quality
33	Ong & Stoner, 2018 [60]	Retrospective Cohort Study	n = 852,111	Patients who had their first lower back pain diagnosis between October 2011 and September 2013 and had a 1- or 2-level lumbar fusion	>18 years old	To evaluate opioid usage patterns for patients with low back pain and spinal fusion surgery	N/A	N/A
34	Owusu-Agyemang et al., 2019 [61]	Retrospective Cohort Study	n = 288	Patients who underwent cytoreductive surgery with hyperthermic intraperitoneal chemotherapy between January 2006 and July 2018 at The University of Texas MD Anderson Cancer Center	>19 years old	To describe the rates of outpatient opioid use postoperatively at 6 and 12 months and to determine which perioperative factors were associated with opioid use at 6 and 12 months	Age	Social and Community Context
35	Patel et al., 2022 [62]	Retrospective Cohort	n = 92	Adults who underwent total knee or hip replacement at the University of Illinois Hospital between August 2019 through March 2020	>18 years old	To evaluate the effect of a standardized postoperative multimodal pain regimen order set on cumulative opioids prescribed postoperatively at discharge	Age, Sex	Social and Community Context
36	Prabhu et al., 2022 [63]	Retrospective Cohort	n = 106	Patients undergoing elective, primary, single, or multi-level cervical disc replacement procedures from one surgeon in an ambulatory surgical center between June 2017 and December 2021	>18 years old	To evaluate the effect of an enhanced multimodal analgesic protocol on patient-reported outcome measures	Age	Social and Community Context
37	Qian et al., 2019 [64]	Prospective Cohort	n = 70	Patients scheduled for otologic surgery at the Stanford Ear Institute between February 2018 and February 2019 who did not have a history of chronic opioid use	>18 years old	To evaluate opioid consumption following outpatient otologic surgery	Age, Sex	Social and Community Context
38	Santos-Parker et al., 2021 [65]	Retrospective Cohort Study	n = 117	Adults with chronic kidney disease who underwent outpatient upper extremity vascular access surgery at a single outpatient surgical center.	42–69 years old	To examine postoperative opioid prescription and use in patients undergoing vascular access surgery where preoperative opioid exposure is common	Age, Sex	Social and Community Context
39	Sim et al., 2019 [66]	Prospective Pilot Study	n = 65	Patients who underwent laparoscopic appendectomy or cholecystectomy at a single academic institution.	~46 years old	To evaluate patients’ adherence to pain control regimen, post-discharge opioid use, and adequacy of pain control	Affordability of medications	Healthcare Access and Quality
40	Thiels et al., 2018 [67]	Prospective Cohort Study	n = 2486	Adults who underwent 1 of 25 elective procedures performed at 1 of 3 academic medical institutions located in Arizona, Minnesota, and Florida.	54–72 years old	To examine postoperative opioid use to inform the development of opioid prescribing guidelines	Age, Sex, Race/Ethnicity	Social and Community Context
41	Waljee et al., 2016 [68]	Retrospective Cohort Study	n = 296,452	Adults who underwent the following upper extremity procedures identified through Truven Health MarketScan Commercial Claims and Encounters.	>18 years old	To examine the use of opioids following outpatient upper extremity procedures	Age, Sex, Income, Insurance Type	Healthcare Access and Quality, Economic Stability, Social and Community Context
42	Young et al., 2020 [69]	Retrospective Cohort Study	n = 888	Adults who underwent trauma evaluation at a single academic medical center.	26–86 years old	To evaluate geriatric opioid prescriptions following a statewide outpatient prescribing limit	Age, Sex, Race, Policy, Access to Healthcare Services	Healthcare Access and Quality, Social and Community Context, Neighborhood and Built Environment
43	Zipple et al., 2019 [70]	Retrospective Cohort study	n = 722	Opioid-naïve adults who underwent the following surgical procedures at a single community hospital.	18–94 years old	To evaluate change in postoperative prescription practices in an independent community-based hospital after hospital interventions and state legislation	Age, Sex, Race, Policy, Access to Healthcare Services	Healthcare Access and Quality, Social and Community Context, Neighborhood and Built Environment

**Table 2:** Variance in prescription rates based on SDoH and types of surgery for postoperative pain.

#	Primary Author/Year	Type of Surgery	Healthcare Setting	Type of Postoperative Pain	Type of Prescription	Findings for Variance in Prescription Rates Based on SDOH
1	Allan et al., 2020 [30]	Laparoscopic cholecystectomy, laparoscopic inguinal hernia repair, laparoscopic umbilical hernia repair, open umbilical hernia repair, and laparoscopic inguinal hernia repair	Academic Hospital	Acute Postoperative Pain	Opioids, NSAIDs, Acetaminophen	N/A
2	As-Sanie et al., 2017 [31]	Hysterectomy	Outpatient Hospital	Acute Postoperative Pain	Opioids	The authors conclude that patients with pre-existing chronic pain and current pain medication use are more likely to consume opioids postsurgically and conclude that a personalized approach to pain management should be developed for postoperative pain
3	Asanad et al., 2020 [32]	Vasectomy	Outpatient Hospital	Acute Postoperative Pain	Opioids, Ibuprofen	N/A
4	Asmaro et al., 2021 [33]	Craniotomy for tumor resection	Academic Hospital	Acute Postoperative Pain	Opioids	N/A
5	Bateman et al., 2017 [34]	Cesarian Section	Academic Hospital	Acute Postoperative Pain	Opioids	N/A
6	Bhashaym et al., 2019 [35]	Arthroscopy/tendoscopy, Neuroma, Hardware removal, Sesamoidectomy, Mass or ganglion resection, Lateral ligament reconstruction, Peroneal tendon repair, Bunion surgery, First MTP fusion/cheilectomy, Forefoot procedures, Fusions (except first MTP), Total ankle replacement, Flatfoot correction, Below-knee amputation Open reduction internal fixation	Outpatient Hospital	Acute Postoperative Pain	Opioids	The authors report that among the independent risk factors for increased opioid consumption were younger age, male sex, and recent preoperative opioid use. Average opioid consumption was highest (27 pills) among patients between 40 and 49 years of age The difference in pill consumption by 10-year age groups demonstrated statistically significant differences (P = 0.003). There was a statistically significant difference in opioid consumption by patient sex (female vs. male = 17 vs. 23 pills, respectively; P = 0.007) The authors found no statistically significant difference in consumption or utilization by insurance type
7	Bronstone et al., 2022 [36]	Outpatient lower extremity orthopedic surgeries (Ankle ORIF, Soft-tissue surgery, Implant removal, Meniscus repair/debridement, ACL reconstruction)	Academic Hospital	Acute Postoperative Pain	Opioids	A high proportion of Black patients and individuals with Medicaid insurance was found in the study, both risk factors for prolonged opioid use after surgery
8	Cooperman et al., 2017 [37]	Middle Ear Surgery and Mastoidectomy	Outpatient Hospital	Acute Postoperative Pain	Opioids	Older patients are prescribed fewer opioids. For every 10-year increase in age, patients received a half tablet less of 5-mg hydrocodone pill equivalents Men were prescribed higher doses than women In comparison with Caucasian patients, Asian patients received smaller opioid prescriptions Increasing income is associated with smaller dosages of opioids in comparison with the lowest income bracket Education level was not associated with morphine milligram equivalents (MME)
9	Cron et al., 2019 [38]	General, Joint/Spine, Oncologic, Cardiothoracic/Vascular, Gynecologic	Teaching and Non-teaching Hospitals	Acute Postoperative Pain	Opioids	N/A
10	Ford et al., 2022 [39]	Bariatric Surgery (sleeve gastrectomy (SG) or Roux-en-Y gastric bypass (RYGB) procedure)	Academic Hospital	Acute Postoperative Pain (Pain experienced in the week after surgery)	Opioids	N/A
11	Fujii et al., 2018 [40]	Specialty (Orthopedic surgery, General surgery, Gynecology, Urology): Outpatient procedures	Academic Hospital	Acute Postoperative Pain	Opioids	N/A
12	Hill et al., 2017 [41]	General Surgery (partial mastectomy, partial mastectomy with sentinel lymph node biopsy, laparoscopic cholecystectomy, laparoscopic inguinal hernia repair, and open inguinal hernia repair)	Academic outpatient surgical center	Acute Postoperative Pain	Opioids	No significant relationship between patient age and the number of opioids prescribed was reported Variations in frequency of provider prescription rates for patients undergoing same surgery was seen, leading to excess opioid use in certain number of patients and increased risk of adverse health outcomes
13	Hite et al., 2023 [42]	Anorectal Procedures	Outpatient Hospital	Acute Postoperative Pain	Opioids	N/A
14	Holst et al., 2020 [43]	Lung resection with either minimally invasive surgery (MIS) or thoracotomy	Academic Centers	Preoperative pain score (pain score in the 30 days leading up to surgery), the maximum pain score (the highest pain score from the day of hospital admission through hospital discharge), and the discharge pain score (last pain score of the hospitalization)	Opioids	N/A
15	Jandali et al., 2020 [44]	Major oral cavity resection, oropharynx resection, laryngectomy, or pharyngectomy	Academic Hospital	Acute Postoperative Pain	Gabapentin, Celecoxib, Acetaminophen, Ketorolac, Fentanyl Patch	N/A
16	Janek et al., 2022 [45]	Dialysis access surgery	Academic Hospital	Acute Postoperative Pain, Chronic Postoperative Pain	Hydrocodone/Acetaminophen, Oxycodone, Oxycodone/Acetaminophen, Tramadol, Codeine, Hydromorphine	N/A
17	Jawad et al., 2022 [46]	Any of the 41 most common otolaryngology procedures in the current procedural terminology code system	Academic Hospital	Acute Postoperative Pain	Hydrocodone/Acetaminophen, Oxycodone/Acetaminophen, Codeine	N/A
18	Katz et al., 2021 [47]	7 common outpatient otolaryngology surgical procedures	Academic Hospital	Acute Postoperative Pain	Opioids	N/A
19	Keller et al., 2021 [48]	Outpatient hemorrhoid, fissure, or fistula procedures	Academic Hospital	Acute Postoperative Pain	Opioids	Patients living in the Western Region of the country exhibited higher rates of new persistent opioid use Patients with Bachelor’s degree or higher had lower rates of new persistent opioid use
20	Kelley et al., 2020 [49]	Urological	Academic Hospital	Acute Postoperative Pain	Opioids	N/A
21	Kim et al., 2016 [50]	Upper extremity orthopedic	Academic Hospital	Acute Postoperative Pain	Oxycodone/Acetaminophen, Acetaminophen/Hydrocodone, Acetaminophen/Codeine	Male patients reported taking higher mean number of pills for a longer period following surgery 30- to 39-year-olds had the highest mean opioid consumption following surgery Patients who self-pay or have Medicaid reported consuming greatest number of opioids
22	Kirkness et al., 2013 [51]	Total hip arthroplasty	Academic Hospital	Acute Postoperative Pain	NSAIDs (celecoxib, Ketorolac, naproxen, meloxicam, ibuprofen); Opioids (tramadol, propoxyphene, codeine, hydrocodone, hydromorphone, meperidine, methadone, morphine, oxycodone, remifentanil, sufentanil)	Opioids were the most commonly prescribed class of pain-related medications both while in hospital and upon discharge, with reliance predominantly on strong opioids as well as celecoxib
23	Kulik et al., 2015 [52]	Coronary artery bypass graft surgery	Database Research	Acute Postoperative Pain	NSAIDs (ibuprofen, indomethacin, naproxen, diclofenac, etodolac, ketorolac, meloxicam, celecoxib, rofecoxib, valdecoxib)	Compared with patients who were not treated with NSAIDs, those who were administered NSAIDs were more commonly men and were significantly younger Of the patients who received NSAIDs after surgery, 89.2% received ketorolac, representing 95.7% of those exposed to NSAIDs on postoperative day 1
24	Letchuman et al., 2022 [53]	Spine surgery	Academic Hospital	Acute Postoperative Pain	Opioids	White patients received higher inpatient and outpatient postoperative opioid dosages compared with Black and Asian patients despite otherwise similar demographic and surgical case characteristics
25	Livingston-Rosanoff et al., 2020 [21]	Outpatient anorectal procedures (fistula surgery, exams under anesthesia, excisional hemorrhoidectomy, incision and drainage, pilonidal excision)	Academic Hospital	Acute Postoperative Pain	Opioids	The majority of patients used less than five opioid pills postoperatively, with patients undergoing fistula procedures, I and Ds, and excisional hemorrhoidectomy using the most On average, most patients reported no or mild postoperative pain
26	Locketz et al., 2019 [54]	Nasal/sinus surgery	Private practice and Academic medical institutions	Acute Postoperative Pain	Opioids, Acetaminophen	A weakly positive but statistically significant correlation was seen between opioid use and postoperative pain; similarly, a weakly positive but significant correlation was seen between postoperative pain and acetaminophen use No meaningful correlation was seen with respect to opioid consumption and use of acetaminophen No significant difference in postoperative pain or opioid consumption was seen with respect to age, sex, specific procedures performed, postoperative steroids, or smoking history
27	Long et al., 2018 [55]	Thyroid and parathyroid surgery	Academic Hospital	Acute Postoperative Pain	Opioids, Acetaminophen	Most patients (97%) were discharged home with an opioid prescription, the majority of which was acetaminophen/oxycodone Patients taking preoperative opioids reported a significantly higher maximum pain score during their hospital stay as compared with opioid-naıve patients Maximum pain scores were significantly higher in patients who received IV acetaminophen postoperatively Lower age, maximum pain score, and preoperative opioid use were independently associated with increased morphine milligram equivalents per day
28	Lutsky et al., 2020 [56]	Hand surgery	Academic outpatient surgical center	Acute Postoperative Pain	Opioids	Patients in the commercial insurance group took more morphine equivalent units intraoperatively and in the recovery room and filled fewer prescriptions postoperatively, which were all statistically significant
29	Matteson et al., 2023 [22]	Gynecologic surgery of any type for benign indications (including hysterectomy of any route, laparoscopy without hysterectomy, pelvic organ prolapse repair, incontinence surgery, hysteroscopy, dilation and curettage, and cervical conization)	Academic Hospital	Acute Postoperative Pain	Opioids	N/A
30	McKinnish et al., 2021 [57]	Cesarean delivery	Academic Hospital	Acute Postoperative Pain	Opioids	No significant difference in opioid consumption between Black and White was reported No significant difference in number of opioids prescribed was reported based on race or at discharge
31	Nouraee et al. 2022 [58]	Total knee or hip arthroplasty	Outpatient surgical center and Inpatient academic hospital	Acute Postoperative Pain	Opioids	Lower number of pills prescribed and consumed significantly differed in the outpatient vs. inpatient setting Postoperative pain was significantly lower for TKA in the outpatient vs. inpatient setting Satisfaction of pain control was significantly higher for TKA in the outpatient vs. inpatient setting
32	O’Sullivan et. al, 2023 [59]	Orthopedic procedures, including rotator cuff repair, shoulder arthroscopy, knee arthroplasty, meniscus repair, knee articular cartilage repair, carpal tunnel repair, total hip arthroplasty, or total knee arthroplasty	Academic Hospital	Acute Postoperative Pain	Opioids	No significant difference in the rates of opioid prescriptions between Black, Hispanic/Latino, or Asian/Pacific Islander patients and non-Hispanic White patients was reported Women were less likely to receive an opioid prescription than men Patients aged 18–34 were more likely to receive an opioid prescription than those aged 70+ Difference in the rates of opioid prescriptions between patients with private insurance and those who self-paid was significant Patients with Medicaid/Medicare insurance were less likely to receive an opioid prescription than patients with private insurance
33	Ong & Stoner, 2018 [60]	Spinal fusion surgery	Research Database	Chronic Postoperative Pain	Opioids	N/A
34	Owusu-Agyemang et al., 2019 [61]	Cytoreductive surgery	Academic Hospital	Chronic Postoperative Pain	Opioids	Patients aged > 55 years had increased odds of opioid use within the 12th postoperative month
35	Patel et al., 2022 [62]	Total knee replacement/Total hip replacement	Academic Hospital	Acute Postoperative pain	Opioids	Female sex, younger age, and history of substance use disorder increased opioid requirements following surgery Improvement in provider education significantly reduced the cumulative number of opioids prescribed
36	Prabhu et al., 2022 [63]	Cervical disc replacement	Outpatient Surgical Center	Acute Postoperative pain	Opioids/Acetaminophen	Average patient in the study was below the age of 50 (46.4) and benefitted from the enhanced multimodal analgesic protocol, leading to reduced opioid prescriptions postoperatively
37	Qian et al., 2019 [64]	Otologic surgery	Academic Hospital	Acute Postoperative pain	Opioids	No difference in opioid prescription rate between males and females No correlation between opioid prescription rate and age
38	Santos-Parker et al., 2021 [65]	Arteriovenous fistula, Basilic vein transposition, Arteriovenous graft	Outpatient surgical center	Acute Postoperative pain	Opioids	N/A
39	Sim et al., 2019 [66]	Cholecystectomy, Appendectomy	Academic Hospital	Acute Postoperative pain	Opioids Ibuprofen Acetaminophen	N/A
40	Thiels et al., 2018 [67]	Carotid endarterectomy, Parathyroidectomy, Arteriovenous fistula, MIS partial colectomy with anastomosis, Carpel tunnel release, MIS cholecystectomy, MIS inguinal hernia repair, Ovarian cancer cytoreduction, Open inguinal hernia repair, Simple mastectomy +- sentinel node, Breast lumpectomy +- sentinel node, MIS hysterectomy, MIS low anterior resection +- diverting ileostomy, MIS prostatectomy, MIS nephrectomy, Knee arthroscopic meniscectomy, Open pancreaticoduodenectomy, MIS lung wedge resection, Tonsillectomy, Rotator cuff surgery, Lumbar laminotomy/laminectomy, Open lung lobectomy, Lumbar fusion, Total hip, Total knee	Academic Hospital	Acute Postoperative pain	Opioids	N/A
41	Waljee et al., 2016 [68]	Carpal tunnel release, trigger finger release, cubital tunnel release, thumb carpometacarpal arthroplasty	Research Database	Acute Postoperative pain	Opioids	Older patients are less likely to receive a refill of opioids Patients 65 years and older were 23% less likely to receive a refill compared to patients aged 18 to 34 years old Patients who resided in areas with the highest median household income were less likely to receive a refill of opioids Patients residing in high-income areas were 43% less likely to receive a refill of opioids than patients in areas of lower median household income
42	Young et al., 2020 [69]	None	Academic Hospital	Acute Postoperative pain	Opioids	Geriatric patients were less likely to be prescribed opioids than non-geriatric patients 35% of non-geriatric patients were prescribed opioids, while only 27% of geriatric patients were prescribed opioids Geriatric patients were more likely to receive a greater quantity of opioids Geriatric patients received an average of 150 morphine equivalent doses (MEDs), while non-geriatric patients only received an average of 113 MEDs
43	Zipple et al., 2019 [70]	Cholecystectomy, Appendectomy, Open inguinal hernia repair, minimally invasive inguinal hernia repair, Lumpectomy	Community Hospital	Acute Postoperative pain	Opioids	No association between age, sex, or race and prescription rate of opioids was reported

N/A: Not applicable.

## Data Availability

No new data were created or analyzed in this study. Data sharing is not applicable to this article.
